# Predictors of Residual Severe Tricuspid Regurgitation After Transcatheter Mitral Valve Repair

**DOI:** 10.1016/j.jscai.2023.100612

**Published:** 2023-05-19

**Authors:** Craig Basman, Arber Kodra, Luigi Pirelli, Ahmad Mustafa, Priti Mehla, Biana Trost, Caroline Ong, Taylor Remillard, Emily Schultz, Denny Wang, Shangyi Liu, Efstathia Mihelis, Bruce Rutkin, Elana Koss, Robert Kalimi, Gregory Maniatis, Azhar Supariwala, S. Jacob Scheinerman, Chad Kliger

**Affiliations:** a Department of Cardiovascular and Thoracic Surgery, Lenox Hill Hospital/Northwell Health, New York, New York; b Department of Cardiovascular and Thoracic Surgery, Staten Island/Northwell Health, New York, NY; c Department of Cardiovascular and Thoracic Surgery, North Shore University/Northwell Health, New York, New York; d Department of Cardiovascular and Thoracic Surgery, South Shore University/Northwell Health, New York, New York

**Keywords:** transcatheter mitral valve repair, transcatheter mitral valve replacement, tricuspid regurgitation, transcatheter edge-to-edge repair

## Abstract

**Background:**

Severe tricuspid regurgitation (TR) may persist after a mitral transcatheter edge-to-edge repair (M-TEER) and is associated with worsened clinical outcomes and survival. It is unclear which patients with concomitant mitral regurgitation (MR) and TR will have TR reduction after M-TEER. The aim of this study was to identify the predictors of residual TR after transcatheter edge-to-edge repair (TEER).

**Methods:**

Data were collected from the Northwell TEER registry, a prospectively maintained mandatory database including 4 high-volume transcatheter aortic valve replacement/TEER centers. Transthoracic echocardiograms, both pre-TEER and post-TEER, were evaluated. Univariate and multivariate logistic regression analyses were performed to identify predictors of severe TR after M-TEER. Significant TR reduction was defined as a reduction in TR grade by at least 1+ with moderate (2+) or less TR at 1 month.

**Results:**

Of the 479 patients who underwent M-TEER, 107 patients with concomitant severe MR/TR were included. Successful MR reduction occurred in 89 patients (84%) and a significant TR reduction in 45 (42%). On the univariate analysis, the only predictors of severe residual TR were right atrial area and unsuccessful M-TEER. On the multivariate logistic regression model, the only predictor variable for patients with a reduction in TR was MR reduction of ≥3+ with M-TEER.

**Conclusions:**

In patients with concomitant severe MR and TR, TR reduction after isolated M-TEER occurs in only ∼40% of patients. MR grade reduction ≥3+ was the only independent predictor for TR reduction. Other clinical and echocardiographic variables (including pulmonary hypertension, right ventricular function, tricuspid annular dilation, atrial fibrillation, and presence of a cardiac implantable electrical device) were not associated with residual TR. Inability to predict TR reduction after M-TEER highlights the importance of establishing transcatheter tricuspid valve therapies and should factor in heart-team discussions.

## Highlights

• Concomitant mitral and tricuspid regurgitation is common among M-TEER patients.
• TR reduction after M-TEER occurs in only ∼40% of patients.
• ≥3+ MR reduction was the only predictor for TR reduction after M-TEER

## Central Illustration

**Figure:**
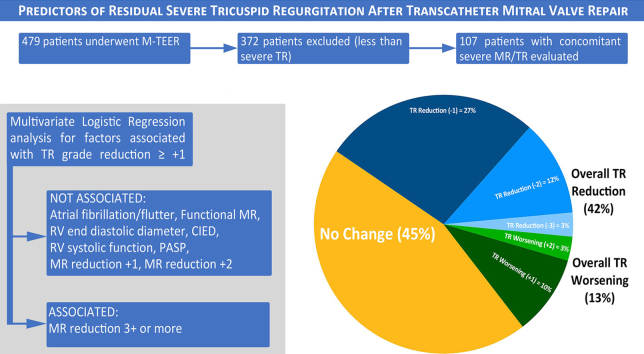

## Introduction

Mitral transcatheter edge-to-edge repair (M-TEER) for mitral regurgitation (MR) is indicated in patients with high surgical risk.^1^ However, currently, there is no recommendation for transcatheter treatment for concomitant tricuspid regurgitation (TR). In the surgical population, current valvular guidelines recommend performing concomitant tricuspid valve (TV) surgery in patients undergoing left-sided valve surgery who also have severe TR, mild-to-moderate TR with dilated annulus (≥40 mm), or signs of right-heart failure.^1^ However, the TV is neglected in patients with concomitant MR and TR who undergo M-TEER. In addition, the presence of moderate-to-severe TR after M-TEER is associated with worsened clinical outcomes and survival.^2^, ^3^, ^4^ Previous studies have shown that TR improves in <50% of patients after transcatheter edge-to-edge repair (TEER).^5^, ^6^, ^7^ Therefore, the ability to predict which patients will have residual TR after M-TEER is crucial for procedural selection. This study aimed to address the predictors of residual TR after M-TEER.

## Methods

**Figure 1:**
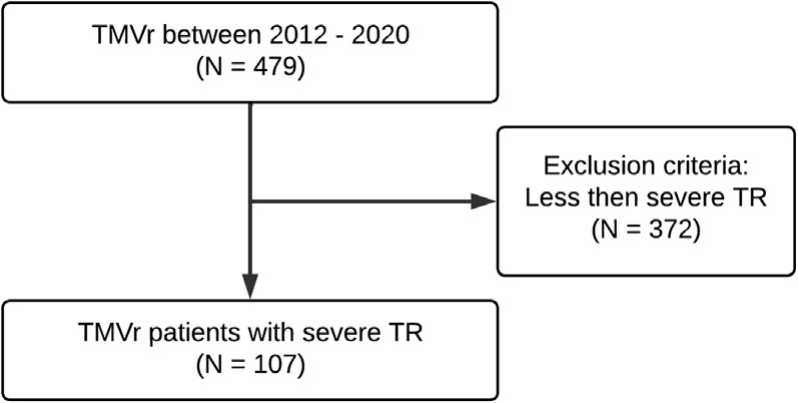
**Schematics of study design.** TMVr, transcatheter mitral valve repair; TR, tricuspid regurgitation.

For this retrospective cohort study, data were collected from the Northwell TEER registry, a prospectively maintained mandatory database including 4 high-volume transcatheter aortic valve replacement/TEER centers. Of the included 479 consecutive patients with symptomatic, moderate-to-severe/severe MR who underwent M-TEER with the MitraClip (Abbott) between 2014 and 2020, 107 patients with concomitant severe MR/TR were analyzed (Figure 1). All patients were evaluated by a multidisciplinary team and deemed to be of high/prohibitive surgical risk. Patients were included if they had 3+ (severe) or more TR before TEER.

### Procedure

Using the MitraClip, M-TEER was performed in a standard fashion.^8^ All TEER procedures were performed under general anesthesia with transesophageal echocardiography guidance.

### Echocardiography

We evaluated the transthoracic echocardiogram (TTE) both pre-TEER and post-TEER. All patients underwent TTE before intervention and at 1-6 months of follow-up. TTE examinations were performed by an imaging cardiologist or echocardiography technician and interpreted by an imaging cardiologist. Six board-certified imaging specialists (C.B., A.K., P.M., B.T., C.O., C.K.) reviewed TTEs pre-TEER and post-TEER, who were blinded to the outcome. TR was assessed in multiple views and graded according to a 5-grade schema: none/trivial = 0+, mild= 1+, moderate = 2+, severe = 3+, massive = 4+, and torrential = 5+.^9^ Left ventricular ejection fraction was calculated in the 2 chamber and 4-chamber views with the Simpson biplane method. Tricuspid annular plane systolic excursion (TAPSE) was obtained. Right ventricular (RV) end-diastolic diameters (or tricuspid annulus diameter [TAD]) were obtained in the 4-chamber view. Pulmonary artery systolic pressure (PASP) was estimated from the TR jet velocity.

### Clinical end points

Patients recorded regular follow-up within 1-6 months and at 1 year after the procedure. The echocardiograms from 1- to 6-month follow-ups were used to assess residual TR (because 1-year echocardiograms were often not obtained). The average echocardiogram follow-up occurred at 1.7 months (median, 1.5 months) after M-TEER. Successful TR reduction was defined as a reduction in TR grade by at least 1+ with moderate (2+) or less TR at 1 month. Data regarding clinical outcomes were obtained from the database that included all clinical end points and echocardiographic parameters.

### Statistical analysis

Categorical variables, presented as counts and/or percentages, were compared using the Fisher exact test. Continuous variables, presented as the mean ± SD or the median (lower quartile, upper quartile), were compared using the Student *t* test or the Mann-Whitney *U* test because the Shapiro-Wilks test showed these variables to be not normally distributed. A *P* value <.05 was considered statistically significant. All statistical tests were 2-tailed and performed using Prism 9.2.0 (GraphPad Software). A multivariable logistic regression model was used to estimate the predictors for severe TR after M-TEER. Clinically important variables such as atrial fibrillation (AF) or flutter, degenerative or functional MR, RV end-diastolic diameter (or TAD), MR improvement, previous cardiac implantable electrical device (CIED) placement, RV function, and PASP were included in the initial multivariable model irrespective of *P* values from the univariate analysis. *P* values of <.1 in the univariate analysis were included in the initial multivariable model. A backward elimination procedure was applied to the initial model, and *P* <.05 criteria were used for variables to stay in the model.

## Results

The average age was 79.2 years with a high Society of Thoracic Surgeons (STS) score (9.35%). Successful M-TEER (MR reduction ≤2+) occurred in 83% of patients. There was a reduction in TR in 45 patients (42%), no change in TR in 48 patients (45%), and worsening of TR in 14 patients (13%). TR reduction of >1 grade occurred in only 15% of patients (Central Illustration).

**Central Illustration:**
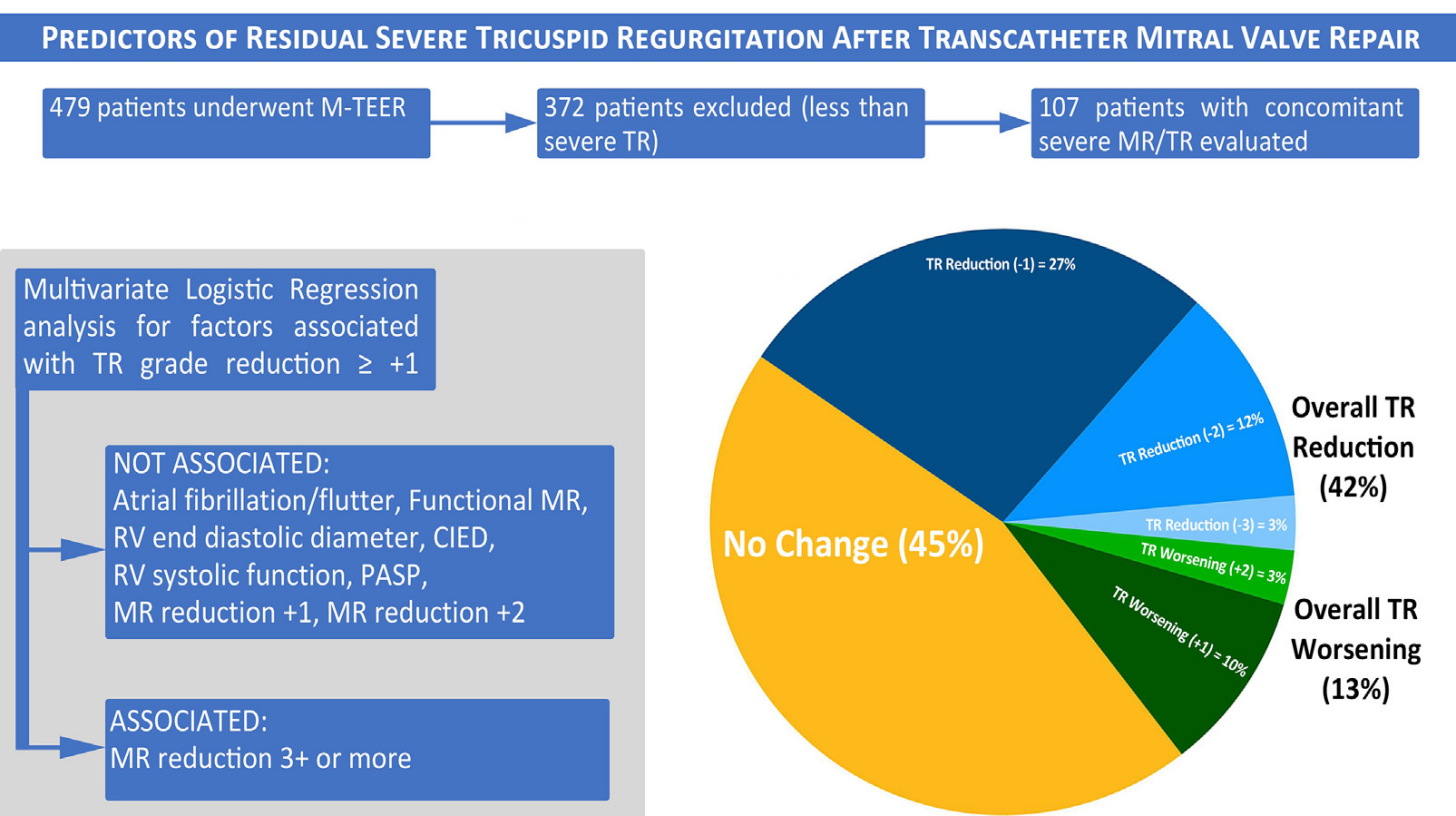
Predictors of residual severe tricuspid regurgitation after transcatheter mitral valve repair. CIED, cardiac implantable electrical device; MR, mitral regurgitation; M-TEER, mitral transcatheter edge-to-edge repair; PASP, pulmonary arterial systolic pressure; TR, tricuspid regurgitation.

The differences between the unsuccessful TR reduction and successful TR reduction groups are described in Table 1. Clinical variables such as age, female sex, body mass index, STS repair score, previous coronary artery bypass grafting, myocardial infarction, AF, left ventricular (LV) systolic dysfunction, hypertension, and presence of a CIED showed no difference between the groups. On the univariate analysis, echocardiographic variables such as RV end-diastolic diameter (TAD), TAPSE, RV end-diastolic area, LV end-diastolic diameter, RV function, inferior vena cava diameter, and etiology of MR (functional vs degenerative) showed no difference between the groups. TR etiology was most commonly functional, with only 8.5% of patients considered to experience a degenerative origin of TR. However, the etiology of TR did not correlate with residual TR. Patients with unsuccessful TR reduction were more likely to record a larger right atrial (RA) area (30.35 cm^2^ vs 24.26 cm^2^; *P* = .002). Among the patients who showed unsuccessful MR reduction with M-TEER, patients were also more likely to experience unsuccessful TR reduction (77.8% vs 22.2%; *P* = .034).

**Table 1:** Univariate predictors of reduction in tricuspid regurgitation after TEER.

Variables	Successful TR reduction (n = 45)	Unsuccessful TR reduction (n = 61)	*P*
Baseline characteristics			
Age, y	78.58 ± 12.08	81.88 ± 9.20	.249
BMI, kg/m^2^	25.25 ± 6.01	25.94 ± 6.41	.464
Female sex	47.92	57.63	.317
STS repair score, %	9.37 ± 10.19	9.34 ± 6.70	.987
CABG	13.56	16.67	.654
Previous MI	15.25	16.67	.842
Hypertension	83.33	94.92	.061
Atrial fibrillation	60.42	72.88	.172
Presence of CIED	20.83	16.95	.608
Left-sided heart variables			
LV systolic dysfunction	34.48	35.42	.920
LV end-diastolic diameter, mm	47.7 ± 9.7	53.2	.270
Functional MR	44.07	45.83	.855
Unsuccessful TEER	22.22	77.78	.034
Right-sided heart variables			
RV dysfunction (moderate or severe)	21.28	25.86	.583
TAPSE, mm	18.23 ± 5.45	17.26 ± 4.56	.177
PASP >50 mm Hg	54.35	38.98	.117
IVC diameter, cm	2.36 ± 2.5	2.19 ± 0.66	.238
TR pathology (degenerative)	8.51	8.47	.995
RV end diastolic diameter, mm	41.25 ± 7.73	43.58 ± 8.34	.267
RA end systolic area, cm^2^	24.23 ± 7.97	30.35 ± 11.21	.003
RV end diastolic area, cm^2^	19.79 ± 5.87	21.94 ± 6.79	.676

Values are mean ± SD or %.

BMI, body mass index; CABG, coronary artery bypass grafting; CIED, cardiac implantable electrical device; IVC, inferior vena cava; LV, left ventricle; MI, myocardial infarction; MR, mitral regurgitation; PASP, pulmonary artery systolic pressure; RA, right atrial; STS, Society of Thoracic Surgeons; TAPSE, tricuspid annular plane systolic excursion; TEER, transcatheter edge-to-edge repair; TR, tricuspid regurgitation.

On the multivariate logistic regression model, the effects of predictor variables for patients with a reduction in TR by 1 grade or more were analyzed. The predictor variables chosen were the presence of CIED, AF/atrial flutter, functional MR, PASP, TAD, moderate/severe RV dysfunction, and MR reduction (by 1, 2, or 3 grades). After the analysis, the only significant predictor for a reduction in TR was an MR reduction of ≥3+ (Table 2).

**Table 2:** Multivariate predictors of reduction in tricuspid regurgitation after TEER.

Clinical variable	Odds ratio	95% CI	*P*
Presence of CIED	0.4939	0.1908-1.225	.1342
Presence of atrial fibrillation/flutter	0.9713	0.3948-2.407	.9494
Functional MR	1.055	0.4349-2.579	.9061
MR reduction by 1+ or less	5.729	0.7016-125.3	.1506
MR reduction by 2+	5.525	0.7973-113.8	.1394
MR reduction by 3+	13.73	1.851-292.0	.0270
Pulmonary artery systolic pressure	1.013	0.9852-1.044	.3651
Tricuspid annular diameter	1.001	0.9424-1.064	.9682
RV function, moderate or worse	0.7304	0.2450-2.095	.5616

CIED, cardiac implantable electrical device; MR, mitral regurgitation; RV, right ventricular.

## Discussion

To date, this study is the largest study evaluating the effect of isolated transcatheter mitral repair on the TV in patients with concomitant severe TR and MR. We found that severe TR not only remains in ∼60% of patients after M-TEER but also worsens in ∼13% of patients. Baseline clinical traits (such as age, presence of CIED, and functional MR) did not correlate with residual TR. The only clinical and/or echocardiographic variables associated with severe residual TR on the univariate analysis were a larger RA area and unsuccessful M-TEER. On the multivariate analysis, we did not identify any preoperative clinical or echocardiographic variables that were predictive of TR reduction after M-TEER (such as CIED, PASP, RV function, functional MR, and TAD). However, patients with a ≥3+ reduction in MR were more likely to experience TR reduction than those with less MR reduction.

In patients who undergo M-TEER, the prevalence of severe residual TR may be >50%.^4^ TR is common after TEER and associated with worse clinical outcomes and survival. Results from the TriValve (Transcatheter Tricuspid Valve Therapies) and TRAMI (Transcatheter Mitral Valve Interventions) registries showed that concomitant transcatheter mitral valve and TV repair was associated with a higher 1-year survival rate than that with isolated TEER in patients with MR/TR.^10^ However, currently, transcatheter TV interventions are not approved in the United States, and few operators have experience with transcatheter mitral valve and TV repair. Previous studies have found that severe TR improves from 20% to 50% in patients who undergo isolated TEER.^5^, ^6^, ^7^

Previously found risk factors for residual TR after M-TEER include advanced age, presence of AF, RV dysfunction, severity of TR, tricuspid annular dilation, and unsuccessful mitral valve repair.^5^^,^^6^ However, this study did not find the presence of AF, TAD, RV dysfunction, and/or advanced age to be factors leading to a lack of improvement in TR. Nonetheless, similar to previous studies, we did find that unsuccessful M-TEER was associated with residual TR. As expected, residual MR may lead to further right-heart stress and a lack of improvement in TV pathology. We also examined the type of MR (degenerative vs functional) and LV dimensions but did not find that baseline pathology of the mitral valve or LV was associated with TR reduction.

This study found that elevated PASP was not associated with residual TR, similar to previous publications.^5^ However, this study did not differentiate the etiology of pulmonary hypertension. Future studies would benefit from, using invasive hemodynamics, evaluating the type of pulmonary hypertension (precapillary, postcapillary, or mixed) and its subsequent association with TR reduction. To our knowledge, this is the first publication to demonstrate a relationship between dilated right atrium with residual TR. The RA size was not evaluated in previous studies.^5^, ^6^, ^7^ The explanation of larger RA size resulting in higher rates of residual TR may imply that atrial functional TR is less likely to improve. It could also mean that the chronicity of TR plays a role, with longstanding TR associated with RA dilation.

Although previous studies found that RV systolic dysfunction increases the chances of residual TR after M-TEER, neither RV dimensions and/or function correlated with residual TR in this study.^11^ RV findings such as RV area, TAPSE, and TAD were also not predictors of TR improvement. These data warrant further investigation with magnetic resonance imaging and/or strain imaging to understand what RV size/function will limit TR improvement. Previous surgical and transcatheter literature has found that larger TAD is less likely to show TR improvement, but this study found that TAD was not associated with residual TR. Future studies will have to examine this relationship, to understand the difference in surgical versus transcatheter TR improvement.

TR has long been associated with the presence of a CIED; however, this study did not find whether patients with a CIED experienced a more residual TR than those without. Theoretically, patients with CIED-mediated TR (ie, lead impingement on the TV) would not show TR improvement despite successful MR reduction.^12^ It is unclear how many patients in this study experienced CIED-mediated TR because the primary focus on transesophageal echocardiography was the mitral valve. Previous publications also found that, after M-TEER, the presence of CIED does not affect TR improvement.^5^, ^6^, ^7^ Therefore, thorough TV imaging is required in such cases to rule out CIED-mediated TR. In cases with no lead impingement, we can expect that TR improvement is as common for patients without CIED.

### Clinical applicability

Reliably predicting which patients with concomitant MR and TR will improve after isolated MR therapy is becoming increasingly important. Current trials are evaluating the use of M-TEER versus surgery in patients with intermediate and low surgical risk: for example, REPAIR MR (NCT04198870) and PRIMARY (NCT05051033). When guidelines expand to offer lower-risk patients transcatheter mitral therapies, it must be considered that TR may not improve. Until transcatheter tricuspid therapies are more established, patients with concomitant TR must then be strongly considered for surgery.

Our findings indicate that the degree of MR reduction was the only reliable predictor of TR reduction. However, it is difficult to anticipate which patients will have unsuccessful M-TEER. With transcatheter mitral valve replacement technologies on the horizon, a greater reduction of MR may be anticipated with replacement devices. If the abolishment of MR is the most effective for TR reduction, isolated MR therapy that yield greater MR reduction (ie, transcatheter mitral valve replacement) may be favored in patients with concomitant MR and TR.

### Limitations

This study was a retrospective study. Six echocardiographic specialists interpreted the TR severity and echocardiographic parameters, so variability among readers may exist. The follow-up period was anywhere from 1 month to 6 months. Most of them were within 2 months, and a longer-term follow-up was poor because many 1-year echocardiograms were not performed. Ideally, a longer-term follow-up would provide more time for remodeling and reassessment of TR. However, previous data show that TR improvement is an early phenomenon (primarily occurring within the first months).^7^

TR is a dynamic process and volume shifts may affect the degree of TR. Follow-up echocardiograms occurred at ∼1 month postoperatively, and volume status of the patients was not reported.

## Conclusion

There are few clinical and echocardiographic predictors of severe TR after M-TEER. Overall, severe residual TR is common after M-TEER and difficult to predict. This study highlights the importance of establishing transcatheter TV therapies for such patients.

## Declaration of competing interest

Chad A. Kliger is a consultant and receives speaking honoraria from Edwards Lifesciences and Medtronic. Luigi Pirelli is a consultant and receives speaking honoraria from Edwards Lifesciences and Medtronic. Azhar Supariwala is a consultant and receives speaking honoraria from Abbott. Bruce Rutkin is a consultant and receives speaking honoraria from Edwards Lifesciences and Medtronic. None of the other authors have anything to disclose.
